# Intravaginal Application of Topical Black Salve for High-Grade Cervical Intraepithelial Neoplasia

**DOI:** 10.31486/toj.19.0044

**Published:** 2020

**Authors:** Peter J. Ayoub, Angela Parise

**Affiliations:** ^1^ The University of Queensland Faculty of Medicine, Ochsner Clinical School, New Orleans, LA; ^2^ Department of Obstetrics and Gynecology, Ochsner Clinic Foundation, New Orleans, LA

**Keywords:** *Administration–intravaginal*, *cervical intraepithelial neoplasia*, *sanguinarine*

## Abstract

**Background:** Black salve, or sanguinarine, is a topical escharotic agent that has been used by patients for homeopathic ablation of epithelial dysplasia, including cervical intraepithelial neoplasia.

**Case Report:** A 33-year-old female presented to the obstetric and gynecologic clinic for management of a missed abortion. At the time of presentation, she admitted to the use of topical black salve for treatment of cervical intraepithelial neoplasia 2 years prior. Speculum examination revealed a stenotic cervix that appeared flush against the vaginal cuff. Hysteroscopy performed 4 months later after the patient developed new oligomenorrhea revealed significant vaginal scarring with formation of a blind pouch that concealed the true cervix.

**Conclusion:** Health care providers should be aware of homeopathic remedies trialed by patients on their own or as an alternative to recommended treatment. Such self-treatment may cause significant patient harm, such as scarring or deformity.

## INTRODUCTION

Black salve is a topical escharotic agent that has received significant attention in the alternative medicine community as a treatment for cancers and many other disease processes.^1,2^ The active ingredient in black salve is sanguinarine, derived from bloodroot (*Sanguinaria canadensis*) and usually prepared in a mixture of zinc chloride.^1,3^ Sanguinarine has been found to produce oxidative stress–dependent cellular apoptosis by compromising mitochondrial integrity and producing apoptogenic caspase proteins.^4^ Because of the potent induction of apoptosis, sanguinarine has received significant attention regarding its potential in medicine.

Black salve can be easily obtained through online purchase and is marketed as a safe and effective treatment for a variety of cutaneous diseases. No clinical trials have been conducted to assess the effectiveness or safety profile of sanguinarine for treatment of neoplastic disease.^2^ Nonetheless, patients continue to use black salve as a topical agent for the treatment of epithelial dysplasia even though the US Food and Drug Administration has not approved it for such use.

Cervical intraepithelial neoplasia (CIN) is a precancerous change to the cervical epithelium that has the potential to progress to cervical cancer, typically during the course of 10 to 20 years.^5^ The World Health Organization recommended treatment options for CIN are cryotherapy, loop electrosurgical excision procedure (LEEP), or cold knife conization (CKC).^6^ LEEP and CKC are widely practiced excisional procedures. Cryotherapy is ablative and therefore comparable to the perceived mechanism of escharotic therapy in that it destroys the neoplastic tissue.

This case provides a unique perspective into the adverse effects of intravaginal application of black salve preparations.

## CASE REPORT

A 33-year-old female gravida 2 para 0110 presented to the obstetric and gynecologic clinic to establish prenatal care. Her estimated gestational age determined by last menstrual period was 10 weeks 6 days. A diagnosis of missed abortion was made after ultrasound revealed a crown rump length of 9.2 mm, consistent with a gestational age of 7 weeks 0 days, with no fetal heartbeat identified.

The patient had a history of normal 28-day menstrual cycles and used a combined oral contraceptive pill prior to this pregnancy. She reported a history of polycystic ovary syndrome, human papillomavirus infection, and CIN grade 3 (CIN 3) diagnosed 3 years prior. Two years prior to the current visit, the patient received cryotherapy treatment for CIN 3 that failed to resolve her cervical dysplasia as evidenced by a repeat Pap smear. She was offered CKC but declined, and instead opted for homeopathic treatment with topical black salve. She reported that her cervical dysplasia was confirmed as resolved by Pap smear after one treatment with black salve; however, she applied another treatment that resulted in significant vaginal pain.

Pelvic examination at presentation revealed normal external genitalia without lesions and normal hair distribution. The vagina was moist and well rugated without lesions or discharge. The vaginal canal appeared shortened, and the cervix appeared pink and flush with the vaginal cuff. The uterus was of normal size, mobile, without tenderness, and with no evidence of adnexal masses or tenderness. She was prescribed oral misoprostol 800 μg for management of missed abortion, and she passed fetal tissue without complication.

Three months after her initial presentation, the patient returned to the clinic with complaints of oligomenorrhea, with her last menstrual period occurring 38 days prior to this visit. She denied any fevers or pelvic pain. The patient was afebrile, and vital signs were normal. Pelvic examination findings were identical to those from the previous examination. A uterine sound was placed through the external cervical os, and purulent material with a small amount of red blood was passed through the cervix. Human chorionic gonadotropin level was <1.2 mIU/mL. Transabdominal ultrasound revealed no sonographic abnormality of the uterus.

Aerobic cultures were collected at the time of examination. Three days later, the cultures grew *Klebsiella pneumoniae* sensitive to ciprofloxacin. The patient was treated with ciprofloxacin 500 mg orally twice daily for 10 days and instructed to return if signs or symptoms of pelvic abscess developed.

The patient returned to clinic 6 weeks later with continuing oligomenorrhea to discuss a management plan. Hysteroscopy was scheduled 5 days later for evaluation and management of cervical stenosis. On the day of the procedure, visual examination revealed a shortened vaginal canal measuring approximately 5 cm in length and a cervix that appeared flush against the vagina. The cervix was serially dilated to accommodate a 5-mm rigid hysteroscope. Once through the dilated os, the hysteroscope entered a blind pouch measuring approximately 3.5 cm in length, and the true cervix was visible on the right anterior vaginal wall (Figure). Two attempts to advance the hysteroscope through the cervical os failed because of the distorted anatomy. The procedure was terminated after the second attempt, and the hysteroscope was withdrawn without complication.

**Figure.**
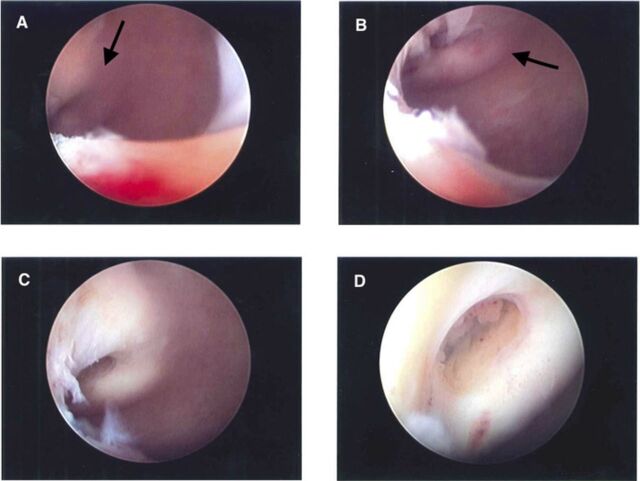
**(A) Hysteroscope image upon entry into the blind pouch shows true cervix (arrow) visible on the right anterior vaginal wall. (B) Hysteroscope is directed at the cervix (arrow) from the entry of the blind pouch. (C) Cervix is clearly visible on the right anterior vaginal wall. (D) Closer view of the cervix shows glandular tissue in the os.**

The patient was referred to the urogynecology service to discuss management of her newly diagnosed vaginal stenosis. Vaginoscopy and hysteroscopy were scheduled for 3 months later. Vaginoscopy findings were consistent with the prior attempted hysteroscopy. Hysteroscopy revealed a normal cervix leading to an arcuate uterus with normal-appearing ostia. Postoperatively, the patient was recommended to use a cervical dilator daily for 5 to 10 minutes. Future plans were to perform a vaginal adhesiolysis and to place an indwelling vaginal stent to relieve the stenosis.

## DISCUSSION

The application of herbal escharotic agents to a lesion causes burning upon contact and eventual sloughing with eschar formation.^7^ Escharotic treatment therefore presents the opportunity for significant scarring of tissue and formation of adhesions in areas of proximate structures. When escharotic agents are applied to the cervix, vaginal canal stenosis can occur as seen in this case. The significant scarring and vaginal canal stenosis caused distortion of the true cervix within the newly formed blind pouch, making hysteroscopy with a rigid hysteroscope difficult. Another potential sequela of vaginal scarring is retention of menstrual products in the blind pouch, resulting in infection and chronic pelvic pain.

A particularly worrisome complication in this case was the possibility that the patient's supposed negative Pap smear after the initial treatment with black salve was a false negative report because of the likelihood that the cervical tissue was not adequately sampled and the tissue sample was from the stenotic vagina.

While patients can easily access black salve preparations, physician monitoring of their use is often absent. Concerns regarding safety, purity, potency, and efficacy of black salve preparations acquired from unknown or unregulated sources are legitimate. The lack of research behind these topical escharotics further compounds the potential for harm to patients seeking perceived natural therapies. Other case reports describe the deleterious effects of self-treatment with topical black salve escharotics.^2,8,9^ To our knowledge, the present case provides a unique example of intravaginal scarring following the use of black salve for CIN that has not yet been described in the literature.

## CONCLUSION

On the internet, misinformation is just as prevalent as accurate information, and black salve is widely advertised online as a miracle cure for a number of diseases, including CIN. Patients may opt out of recommended therapy in favor of a treatment perceived to be a more natural therapeutic option. Physicians need to be aware of the consequences of potential herbal remedies not supported by legitimate research and to urge inquisitive patients to be cautious. Sanguinarine may have a future in medicine; however, substantial research is needed regarding its mechanism of action and safety profile.

## Notes

This article meets the Accreditation Council for Graduate Medical Education and the American Board of Medical Specialties Maintenance of Certification competencies for Patient Care and Medical Knowledge.
